# Voluntary wheel running behaviour as a tool to assess the severity in a mouse pancreatic cancer model

**DOI:** 10.1371/journal.pone.0261662

**Published:** 2021-12-23

**Authors:** Nora Weegh, Eva Zentrich, Dietmar Zechner, Birgitta Struve, Laura Wassermann, Steven Roger Talbot, Simone Kumstel, Miriam Heider, Brigitte Vollmar, André Bleich, Christine Häger

**Affiliations:** 1 Institute for Laboratory Animal Science, Hannover Medical School, Hannover, Germany; 2 Rudolf-Zenker-Institute of Experimental Surgery, University Medical Center, Rostock, Germany; Belgrade University Faculty of Medicine, SERBIA

## Abstract

Laboratory animals frequently undergo routine experimental procedures such as handling, restraining and injections. However, as a known source of stress, these procedures potentially impact study outcome and data quality. In the present study, we, therefore, performed an evidence-based severity assessment of experimental procedures used in a pancreatic cancer model including surgical tumour induction and subsequent chemotherapeutic treatment via repeated intraperitoneal injections. Cancer cell injection into the pancreas was performed during a laparotomy under general anaesthesia. After a four-day recovery phase, mice received either drug treatment (galloflavin and metformin) or the respective vehicle substances via daily intraperitoneal injections. In addition to clinical scoring, an automated home-cage monitoring system was used to assess voluntary wheel running (VWR) behaviour as an indicator of impaired well-being. After surgery, slightly elevated clinical scores and minimal body weight reductions, but significantly decreased VWR behaviour were observed. During therapy, body weight declined in response to chemotherapy, but not after vehicle substance injection, while VWR activity was decreased in both cases. VWR behaviour differed between treatment groups and revealed altered nightly activity patterns. In summary, by monitoring VWR a high impact of repeated injections on the well-being of mice was revealed and substance effects on well-being were distinguishable. However, no differences in tumour growth between treatment groups were observed. This might be due to the severity of the procedures uncovered in this study, as exaggerated stress responses are potentially confounding factors in preclinical studies. Finally, VWR was a more sensitive indicator of impairment than clinical scoring in this model.

## Introduction

In animal models for pancreatic cancer, surgical interventions for orthotopic injection of cancer cells as well as frequent handling procedures for the application of drugs are indispensable [1], e.g. to investigate systemic influences such as diabetes [2] or the efficacy of drugs [3]. It has, however, sufficiently been demonstrated that stress due to laboratory routines [4] may adversely affect study outcomes, e.g. increased tumour growth [4, 5] or alter the metabolism and immune system [4]. Therefore, experimental procedures should be evaluated with regard to their effect on the well-being of animals by using evidence-based methods, not only ensuring animal welfare but also improving quality of scientific data.

In this context, automated home-cage monitoring becomes increasingly important as it provides continuous monitoring of the animals’ activity without human intervention [6], preventing stressful situations such as transfers to new environments to conduct behavioural tests [7]. This results in higher reproducibility [7, 8] and contributes to experimental refinement [7]. Different methods and tools for automated home-cage monitoring such as telemetry [9], radiofrequency identification (RFID) [10], video recording [11], piezoelectric sensors [12], passive infrared motion sensors [13] or voluntary wheel running (VWR) are already available (for review: [8, 14]). Recently, our group identified VWR behaviour as a robust indicator of disturbed well-being in a mouse colitis model and after restraint stress [15, 16]. In addition, VWR has been used to evaluate the recovery after surgical intervention for partial hepatectomy [17] and to differentiate between varying levels of severity in a study implanting differently sized transmitters [18]. Furthermore, VWR has been utilized as a tool to measure inflammatory pain during peripheral inflammation [19].

In the present preclinical study the effect of surgical pancreatic cancer induction and repeated intraperitoneal injections of galloflavin and metformin or respective vehicle substances on the well-being of mice was investigated. Galloflavin is a lactate dehydrogenase inhibitor [20] leading to the inhibition of cancer growth or death of cancer cells [21–23]. Metformin is usually applied to treat type 2 diabetes mellitus, but also has anti-cancer effects [24] and has been evaluated as an adequate addition to chemotherapy [25] for pancreatic cancer [26]. To assess the severity of procedures for surgical cancer induction and subsequent chemotherapeutic treatment by repeated intraperitoneal (IP) injections, home-cage monitoring of VWR, supplemented by clinical scoring and survey of body weight (BW) was utilized.

## Materials and methods

### Project authorisation

The experiments were conducted with the approval of the State Office of Agriculture, Food Safety and Fisheries Mecklenburg-Western Pomerania (LALLF, license 7221.3-1-019/15-10) and the Lower Saxony State Office for Consumer Protection and Food Safety (LAVES, license 33.8-42502-04-18/2852). All procedures were carried out in accordance with the German law for animal protection and the European Directive 2010/63/EU.

### Animals and husbandry

Twenty male C57BL/6J mice were obtained from the central animal facility of the Hannover Medical School (Hannover, Germany), at the age of 10 weeks. In this study male C57BL/6J mice were used (as in previous studies [26, 27]) to facilitate the comparability of results. All animals were housed separately in EU type II Macrolon^®^ cages (Tecniplast) with autoclaved tap water and standard rodent chow (Altromin 1324, Lage, Germany) supplied ad libitum. Bedding (poplar wood, AB 368P, AsBe-wood GmbH, Germany) was changed once a week with transfer of used material to the new cage to diminish the effects of a new environment. The animal room was kept under a constant 14/10 light-dark cycle (7:00 AM- 9:00 PM) with a room temperature of 22 ± 2°C and a humidity of 55% ± 5%. Routine health surveillance according to Mähler et al. [28] was performed via a sentinel system, revealing the presence of *Pasteurellaceae*. All handling procedures were confined to three experienced staff members and were conducted between one and three hours after lights-on (8:00 AM to 11:00 AM).

### Study design

**Fig 1:**
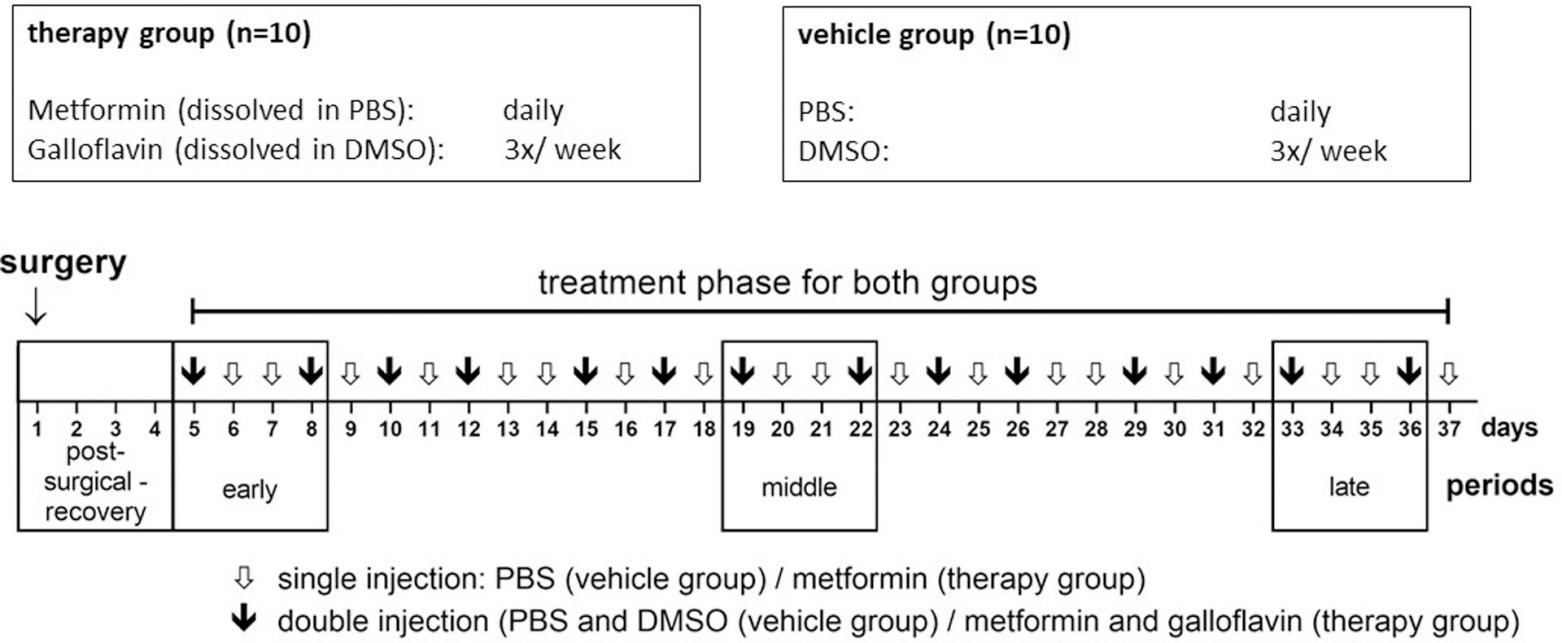
Experimental timeline. Black frames (“□”) indicate different periods (post-surgery, early, middle, late) as well as respective days of subsequent analysis; arrows indicate single (white arrow) and double (black arrow) injection days.

Mice were randomly assigned to a therapy or vehicle group (n = 10 per group) by blindly shuffling and distributing animal score sheets before the start of adaption. The animals were given a total of two weeks to acclimate to the environment and the provided running wheels. Animals underwent surgery for ductal pancreatic adenocarcinoma cell injection on day 1. From day 5 onwards mice of the therapy group received metformin (125 mg/kg, dissolved in 0.1 ml PBS) and galloflavin (20 mg/kg, dissolved in 0.03 ml DMSO). Animals of the vehicle group received vehicle substances PBS (0.1 ml) and DMSO (0.03 ml) on respective days (Fig 1).

Before administration, metformin (1,1 Dimethylbiguanide hydrochloride, Lot # BCBT7573, Sigma-Aldrich Chemie GmbH, Steinheim, Germany) was dissolved in PBS (PBS Dulbecco, Merck Biochrom GmbH, Berlin, Germany) and kept in small aliquots at -20°C with a concentration of 125 mg/ml. After thawing, metformin was kept at 4°C for a maximum of three days. Galloflavin (Galloflavin Potassium salt, Batch #1, Tocris Bioscience, Bristol, UK) was dissolved in 100% DMSO (Dimethylsulfoxide ≥99.5%, Bio-Science Grade, Carl Roth GmbH&Co. KG, Karlsruhe, Germany) with a concentration of 20 mg/ml and kept at -20°C at all times.

All substances were warmed to room temperature and applied via IP injection. Starting on day 5, metformin and PBS were administered every day at two hours after lights-on. Additionally, galloflavin and DMSO were given three times a week (Mondays, Wednesdays and Fridays, see also Fig 1), one hour after PBS/ metformin injection. Hence, animals received two IP injections on Mondays, Wednesdays and Fridays and one IP injection on all other days of the week. At the end of the experiment, animals were sacrificed by CO_2_ inhalation and submitted to cardiac puncture for blood collection. During necropsy, pancreatic tumours were removed from the animals and cleaned of any adhesive tissue before all tumours were weighed. All animals were examined macroscopically for scattered cancer tissue and other pathological changes.

### Tumour cell injection

All 20 animals were injected with ductal pancreatic adenocarcinoma cells (6606PDA cell line, originally a gift of Prof. Tuveson, University of Cambridge, UK) into the pancreas on day 1. Cells were cultured and prepared for injection as previously described [2, 27]. Inhalation anaesthesia was induced with 1 l/min O_2_ and 4 vol% isoflurane in a mouse-sized translucent induction box. After ceasing of righting reflex, mice were placed in a supine position with the nose inside the inhalation mask; the eyes were protected by Bepanthen^®^ eye ointment. Anaesthesia was maintained with 1 l/min O_2_ and 1.5–1.9 vol% isoflurane. The chest and abdominal area were shaved, and a transversal cut was made across the cranial abdomen for opening the skin and abdominal cavity. The pancreas was carefully extracted using cotton swabs and 5 μl of the cooled cell lysate (containing 2.5x10^5^ cells) were injected using a pre-cooled Hamilton syringe. The syringe was drawn back 20 seconds after injection and vesicle formation and location were carefully checked. The wound was closed using Vicryl 5–0 absorbable suture for closure of the muscle and peritoneal layer (continuous suture) and Prolene 5–0 for the closure of the skin (U sutures).

For analgesia, carprofen (Rimadyl, Zoetis Deutschland GmbH, Berlin, Germany) was given once subcutaneously (5 mg/kg body weight) before surgery as well as under anaesthesia. Immediately after surgery, metamizole (Novaminsulfon 500 mg Lichtenstein, Zentiva Pharma GmbH, Frankfurt am Main, Germany) was provided via the drinking water with a concentration of 1250 mg/l throughout the whole experiment.

### Clinical score

All mice were scored daily using a clinical score (Table 1), modified according to Kumstel et al. [29]. On the one hand, the general conditions of the animals were assessed, and on the other hand, the spontaneous and provoked behaviour as well as the process-specific criteria were evaluated.

**Table 1:** Distress score modified according to Kumstel et al. [29].

	**I General condition**	score
I—a	fur dull, ruffled or untended	2
I—b	eyes dull or squinted	2
I—c	pathological discharge from body orifices	3
I—d	abnormal posture (hunched, arched back)	3
I—e	dehydration	3
I—f	short spasms or temporary paralysis symptoms ***or***	3
I—g	longer (>30 seconds) persistent cramping or paralysis	5
I—h	abnormal respiratory sounds or the animal feels cold	5
	**II Spontaneous behaviour**	
II—a	the animal is passive or overactive ***or***	2
II—b	pronounced apathy, hyperkinetic or isolation	4
II—c	spontaneous vocalisation	5
II—d	self-mutilation	5
	**III Provoked behaviour**	
III—a	animal is passive or overactive ***or***	2
III—b	distinct apathy or hyperkinetic	5
	**IV Process-specific criteria**	
IV—a	wound healing disorder	2
IV—b	local inflammation	2
IV—c	ascites	4
	**total score**	**0–50**

### Body weight

For daily body weighing animals were removed from the cage by cup handling and placed within a plastic box on a scale (CM 320-1N, 0,1-320g, KERN & SOHN GmbH Balingen, Germany). Baseline body weights were determined from the last three days of the adaption period and all changes were presented in % as relative change from baseline.

### Wheel running system

For the assessment of individual VWR activity, mice were single-housed throughout the experiment with a freely accessible running wheel (Ø = 11.5 cm) installed in their home cage (setup “Revolyzer-3TS”, preclinics, Potsdam, Germany). A period of 14 days was given for adaption to the wheels. Wheel rotations were recorded by specialised software (DASY Lab 11.0, National Instruments Germany GmbH, Munich, Germany) in one-minute intervals. For the generation of a VWR baseline, data from the last six days of the adaption period were averaged and set as 100% baseline and changes were presented as relative change from baseline.

Readout parameter in this experiment was the total number of rotations in the dark phase. To differentiate between treatments and assess long-term changes, an early (day 5 to 8), middle (day 19 to 22) and a late (day 33 to 36) period were chosen to analyse VWR data (early, middle and late; see also Fig 1). With each period lasting from Friday to Monday, consistency of experimental and environmental influences was maintained. VWR data of these periods were split into two groups according to the substance (therapy or vehicle). These groups were subdivided into two groups each, according to injection frequency (single or double injection). Identification of these four subgroups enabled detection of differences between all four treatment constellations.

To display changes in the activity patterns, VWR data, including the baseline activity on days -6 to -1, were presented as heat maps with each line representing one day. For each day, the values of 5-minute intervals (see S2 File) were summarized for each group and colour-coded with blue representing low and red representing high VWR activity.

### Statistical analysis

All statistical tests were performed using GraphPad Prism^®^ (v8.2.1, GraphPad Software, Inc., La Jolla, CA, USA) and are in detail supplied in S1 File. Microsoft^®^ Excel^®^ (v14.0.7237.5000 Microsoft Office Professional Plus 2010, ^©^Microsoft Corporation) was used to create heat maps for activity pattern analysis. Due to the termination of the experiment for one animal, the vehicle group was reduced to n = 9 animals from day 9 on. A *p*-value of < 0.05 was considered significant in all analyses. Data are depicted as mean ± standard deviation (SD).

To test VWR and body weight change within one group against the baseline, one-way repeated measures (RM) ANOVA using a linear mixed-effects model with restricted maximum likelihood method and Geisser-Greenhouse correction for sphericity control was applied. Animals were treated as random effects, while days and treatment were treated as fixed effects. Model assumptions (normally distributed residuals) were verified in a QQ plot of the actual vs the predicted residuals of the model. Post-hoc testing was performed using Dunnett’s test with baseline data as controls. For daily comparisons between vehicle and therapy group, a two-way RM ANOVA using a linear mixed-effects model was applied (as described above for testing against baseline), using the Bonferroni adjustment for multiple comparisons.

The analysis of the three representative periods (early, middle and late) was performed with a two-way ANOVA with subsequent Tukey’s test for multiple comparisons.

For the analysis of the clinical score or the tumour weight data, a Wilcoxon-signed rank test was performed for baseline comparisons. For comparisons between groups, a Mann-Whitney U test was performed for each day; p-values were Šidák-adjusted.

#### Parameter sensitivity

Receiver operating characteristic (ROC) curves are used in the parameter-free evaluation of binary classifiers. The curve represents the changing trade-off between the false-positive rate (1-specificity, x-axis) and the true positive rate (sensitivity, y-axis) of a discriminator at different values of the cut-off threshold. The binary classes were defined for both, VWR and body weight, and according to two conditions: a) control (as the last day of the adaptation period; class 0) and b) severity (values after surgery; class 1). Here, and at the given order of data points, the resulting ROC curve is an expression of how different both classes are in terms of sensitivity and specificity. An area under the ROC curve (AUC) of 1 indicates an ideal classifier. A high AUC would indicate that by measuring the corresponding variable, either class 0 or 1 can be predicted at high accuracy. The closer the ROC curve is to the 45° line in the plot, the less discriminatory power there is between the states of severity regarding the measured variable. In a second analysis, values of day 4 (class 0) were compared to values of day 5 (class 1) to evaluate the sensitivity of VWR and body weight in response to the influence of the first IP injections.

#### Cluster model

For analyses of post-surgical data (day 1 to 4) and all days of the early, middle and late period, body weight and VWR data were tested in a previously published severity cluster model [15]. The cluster model was developed by using a *k*-means algorithm on experimental training data, resulting in the definition of two borders at 87.37% and 50.16% of baseline VWR. Therefore, it allowed the allocation of individual VWR data to one of three levels (0: < 50.16% VWR; 1: 50.16% to 87.37% VWR; or 2: > 87.37% VWR).

## Results

### Clinical scoring and body weight monitoring during baseline and post-operative recovery

**Fig 2:**
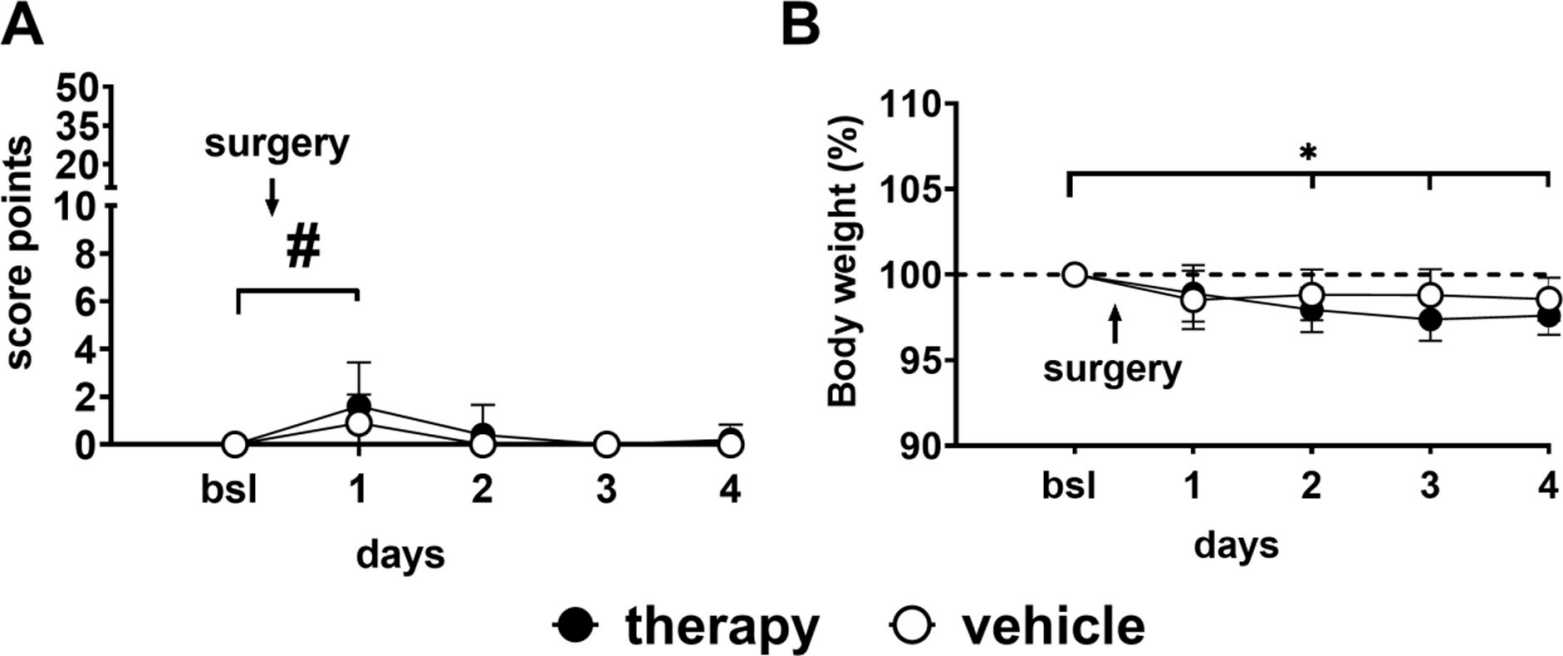
Post-operative recovery after tumour induction. Mice of the therapy and vehicle group (n = 10 each) were clinically scored after surgery. The therapy group showed significantly elevated scores compared to baseline (bsl) merely at day 1 (A; # = therapy; Wilcoxon signed-rank test, *p* < 0.05), and a body weight reduction of up to 2.6% (day 3) on average (B; * = therapy group: linear mixed-effects model, fixed effects type III: *p* < 0.0001, F (6.136, 55.22) = 9.780 with Dunnett’s multiple comparisons test, day 2 to 4, *p* < 0.05).

For the induction of pancreatic cancer, mice were subjected to laparotomy for tumour cell injection into the pancreas under general isoflurane anaesthesia on day 1. The clinical score indicated significant, but only mild signs of impaired well-being on day 1. This was demonstrated by a slight increase of the score up to a maximum of 6 points out of 50 in one animal, but a mean of 1.6 (*p*<0.05) in the therapy group and 0.9 in the vehicle group (#; Fig 2A and S1 File). The animals demonstrated only slight body weight reductions, with highest reductions on day 3 in the therapy group (2.6%) and day 1 in the vehicle group (1.5%) (Fig 2B and S1 File).

### Automated home-cage monitoring of VWR during post-operative recovery

**Fig 3:**
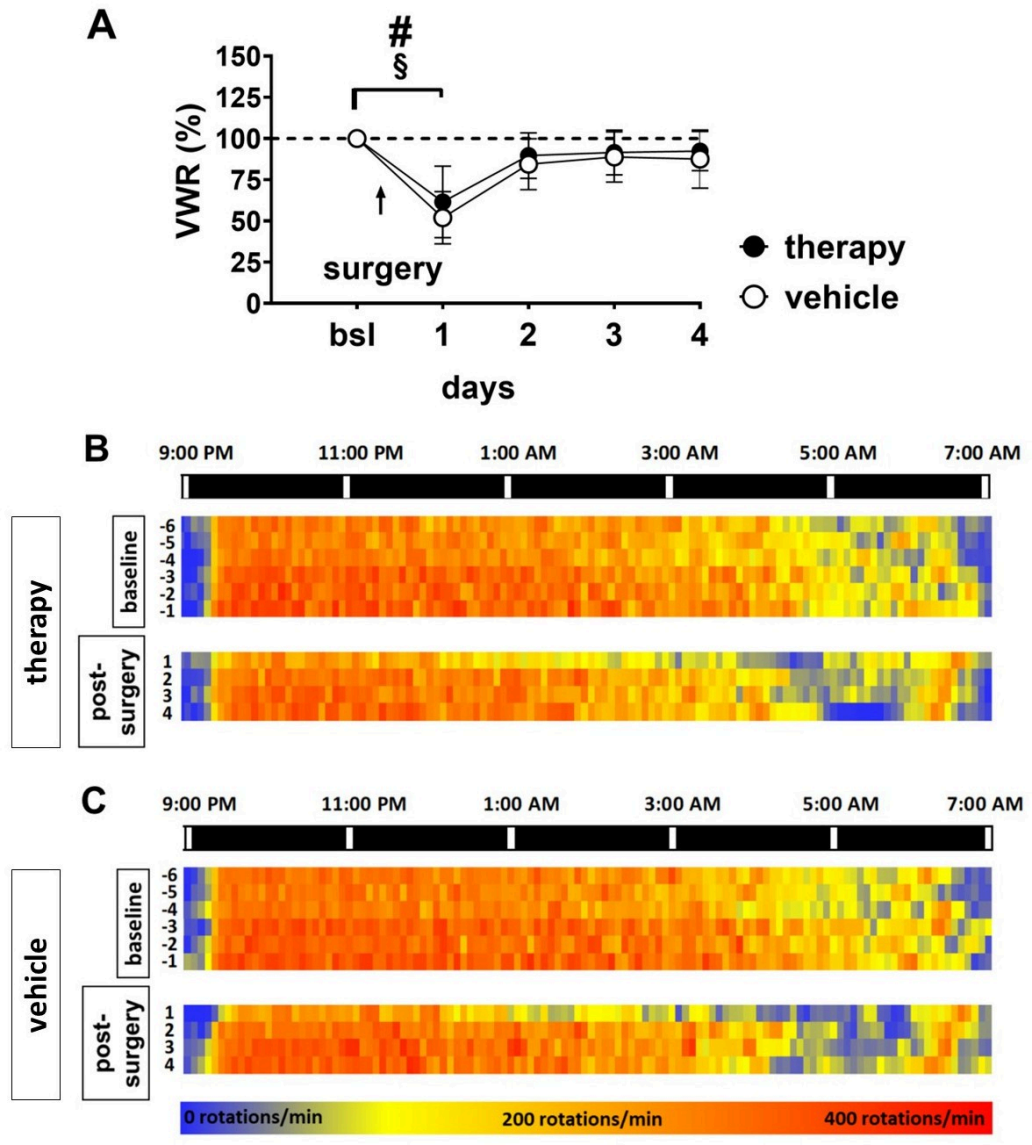
Activity patterns of baseline and post-surgical recovery phase. VWR activity dropped to 62% of baseline in the therapy and 52% in the vehicle group on day 1, followed by an increase to ~90% and ~84%, respectively, on day 2 (A; # = therapy group: linear mixed-effects model, fixed effects type III: *p* < 0.0001, F (3.988, 35.90) = 13.12; Dunnett’s multiple comparisons test *p* = 0.0055; § = vehicle group: linear mixed-effects model, fixed effects type III: *p* < 0.0092, F (3.922, 32.12) = 4.068, Dunnett’s multiple comparisons test *p* = 0.0001). The heat map (B,C) presents the VWR activity data displayed in 5-minute intervals during night-time for days of baseline (day -6 to -1) and post-surgery phase (day1 to 4). This is shown separately for the therapy group (3B) and the vehicle group (3C). Each line represents one dark phase (9 PM-7 AM). For each day, the values of 5-minute intervals are summarized for each group and colour-coded with blue representing low and red representing high VWR activity (0–400 rotations/min). Comparing baseline activity to post-surgical activity a marked reduction can be observed primarily in the second half of the dark phase in both groups.

Monitoring of VWR behaviour revealed a significant drop of activity on day 1 after surgery to a mean of 62% in the therapy group and 52% in the vehicle group (Fig 3A and S1 File). This drop was followed by an increase up to 90% on the next day in the therapy group and to 84% in the vehicle group, remaining on this level until day 4 (Fig 3A).

For a more detailed analysis of VWR behaviour, day- and night-time activity patterns were assessed. Heat maps of baseline activity patterns before surgery revealed very low activity during the light phase (S1 and S2 Figs) and high activity immediately after the onset of the dark phase in both groups (Fig 3B and 3C). This was followed by a decrease in the second half of the dark phase (after six to eight hours) and another increase approximately one hour before the beginning of the light phase (Fig 3B and 3C).

On the day of surgery (d1), mice of both groups demonstrated a shorter period of dark-phase activity (three to four hours after the onset of the dark phase) followed by a longer period of resting and a second increase approximately one hour before the beginning of the light phase (Fig 3B and 3C). Over the following three days, the activity pattern re-adjusted to baseline patterns.

### Clinical scoring and body weight monitoring during chemotherapy and vehicle treatment

In the second part of the study, clinical status and body weight were assessed during chemotherapy and vehicle treatment, which started after surgery on day 5 of the experiment (see also Fig 1).

**Fig 4:**
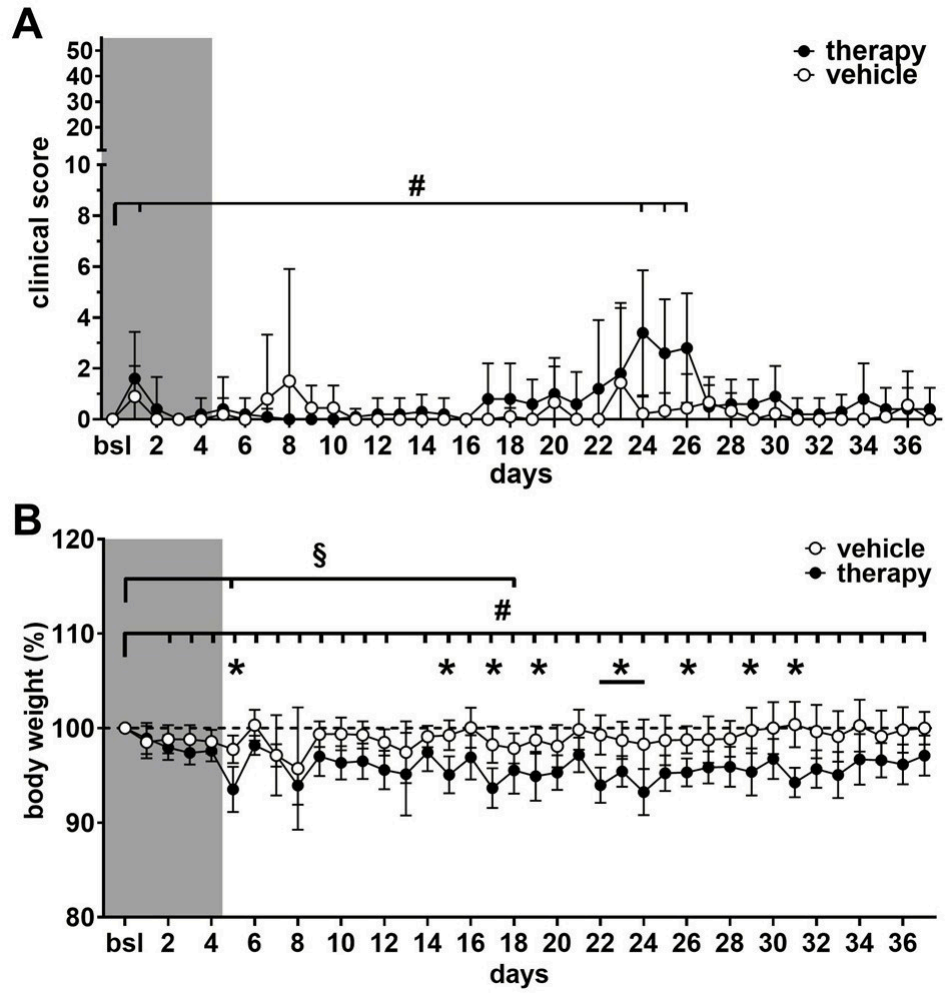
Assessment of clinical score and body weight during chemotherapy and respective vehicle application. Clinical scores and body weight were assessed in therapy (n = 10) and vehicle group (n = 9–10). (A) The course of the clinical score showed slight, although significant increases in the therapy group compared to baseline (#; Wilcoxon signed-rank test, *p* < 0.05) and nodifferences between groups. (B) Body weight was reduced in the therapy group during all but one days in the treatment phase compared to baseline levels (# = therapy group: linear mixed-effects model, fixed effects type III: *p* < 0.0001, F (6.136, 55.22) = 9.780 with Dunnett’s multiple comparisons test, *p* < 0.05). Body weight in the vehicle group was significantly reduced merely on day 5 and 18 (B; § = vehicle group: linear mixed-effects model, fixed effects type III: *p* = 0.0528, F (3.429, 28,08) = 2.785 with Dunnett’s multiple comparisons test *p* < 0.05). Between therapy and vehicle group significant differences in body weight were detected on several days (B; *)(linear mixed-effects model, fixed effects (type III) for group: *p* = 0.0041, F (1, 18) = 10.83 with Bonferroni‘s multiple comparisons test *p* < 0.05).

In mice of the vehicle group, the mean clinical score remained around the baseline level during the entire experiment. In the therapy group, a significantly increased mean score of 3.4 (out of a maximum of 50 score points) was observed on day 24. It remained increased until day 26 due to slightly ruffled fur, squinted eyes and minimal reduction of activity (Fig 4A and S1 File).

The body weight of the vehicle group ranged around baseline levels over the whole observation period (99 ± 1.6%) without further weight gain and was only significantly reduced compared to baseline on day 5 and 18 (Fig 4B and S1 File). Mice of the therapy group showed a more variable course of body weight and did not recover to baseline, remaining at a reduced averageof ~96% of baseline until the end of the experiment. When compared to baseline body weight was significantly reduced on all days, except on day 13 (Fig 4B and S1 File). Body weight differed significantly between therapy and vehicle group on nine out of fourteen double injection days but only on one single injection day (Fig 4B and S1 File).

### Automated home-cage monitoring of VWR during chemotherapy and vehicle treatment

**Fig 5:**
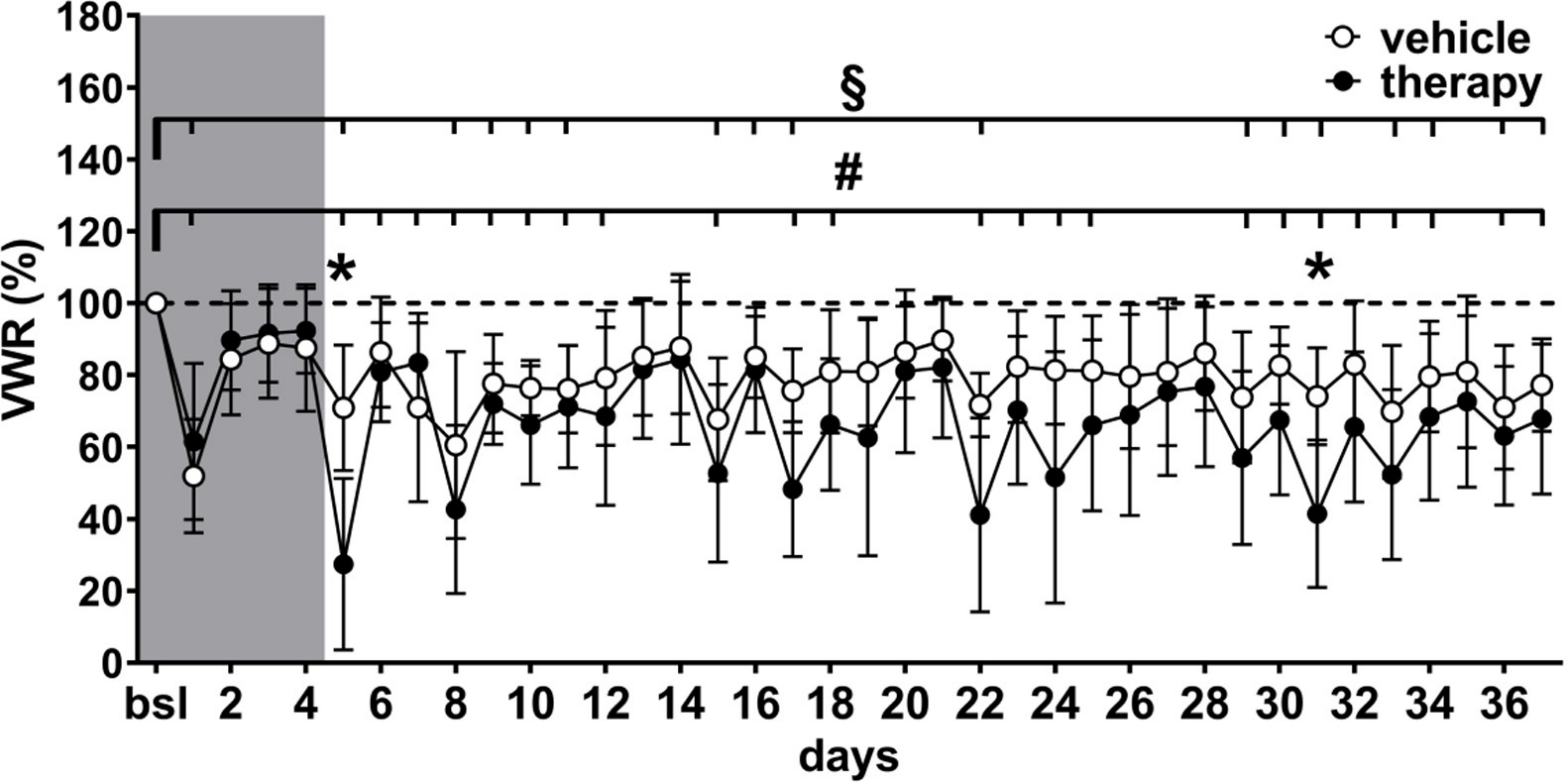
Course of VWR. VWR activity with days of significant differences between groups (day 5 and 31; * = comparison between both groups, linear mixed-effects model, fixed effects (type III) for group: *p* = 0.0927, F (1, 18) = 3.157 with Bonferroni‘s multiple comparisons test *p* < 0.05); compared to baseline, differences were detected in both groups, however, the frequency was higher in the therapy group (# = therapy, linear mixed-effects model, fixed effects type III: *p* < 0.0001, F (3.988, 35.90) = 13.12, Dunnett’s multiple comparisons test *p* < 0.05; § = vehicle, linear mixed-effects model, fixed effects type III: *p* < 0.0092, F (3.922, 32.12) = 4.068 Dunnett’s multiple comparisons test *p* < 0.05).

On the first day of double injections with drugs or vehicle substances a reduction of VWR activity to ~27% of baseline in the therapy group and to 71% of baseline in the vehicle group was observed (Fig 5 and S1 File). On days 6 and 7, VWR activity increased to ~82% of baseline in the therapy group and to ~79% of baseline in the vehicle group. No other significant differences between groups were detectable except on day 31 (Fig 5 and S1 File). However, compared to baseline, significant differences were detected on 12 out of 14 double injection days in the therapy group and 10 out of 14 double injection days in the vehicle group. Additionally, VWR was significantly reduced in both groups on several single injection days (Fig 5 and S1 File).

**Fig 6:**
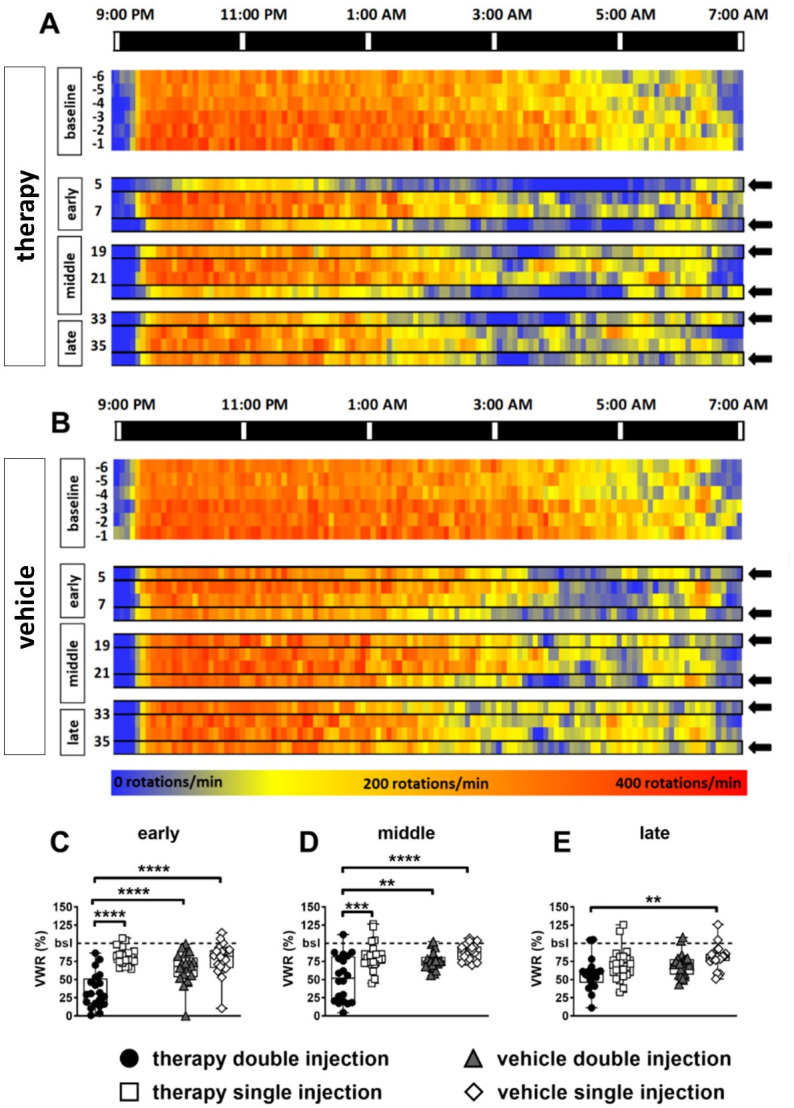
Assessment of VWR during chemotherapy and respective vehicle application. Heat map of VWR activity data displayed in 5-minute intervals during night-time for days of baseline (day -6 to -1) and three representative time periods (early day 5 to 8, middle day 19 to 22, late day 32 to 36), shown separately for the therapy group (6A) and the vehicle group (6B). The arrows indicate the days of double injection. Statistical analysis of the three time periods: (C) early period: RM two-way ANOVA: F (1, 76) = 41.46, *p* < 0.0001 for injection-frequency-dependent variation; F (1, 76) = 8.567, *p* = 0.0156 for group-dependent variation;Tukey’s multiple comparisons test, *p* < 0.0001. (D) intermediate period: RM two-way ANOVA: F (1, 72) = 18.40, *p* < 0.0001 for injection-frequency-dependent variation, F (1, 72) = 10.19, *p* = 0.0021 for group-dependent variation; Tukey’s multiple comparisons test, *p* < 0.01. (E); late period: RM two-way ANOVA: F (1, 72) = 5.889, *p* = 0.0177 for injection-frequency-, F (1, 72) = 5.795, *p* = 0.0186 for group-dependent variation; Tukey’s multiple comparisons test, *p* < 0.01). * = *p* < 0.05, ** = *p* < 0.01, *** = *p* < 0.001, **** = *p* < 0.0001; bsl = baseline.

The heat-map of the activity pattern revealed that the nightly decrease in VWR activity occurred earlier under therapy than during baseline measurements in both groups (Fig 6A and 6B). This drop was especially obvious on the days of double injections and more pronounced in the therapy compared to the vehicle group (Fig 6A and 6B). The statistical analysis of the three representative periods early (day 5 to 8), middle (day 19 to 22) and late (day 32 to 36) revealed differences between treatment groups, indicating a higher drop after application of the therapy substances galloflavin and metformin (Fig 6C–6E). In the early and middle period significant differences were detected between the double-injected therapy group and all other groups. In the late period a significant difference was observed only between animals of the double-injected therapy and the single-injected vehicle group.

### Evaluation of parameter sensitivity

**Fig 7:**
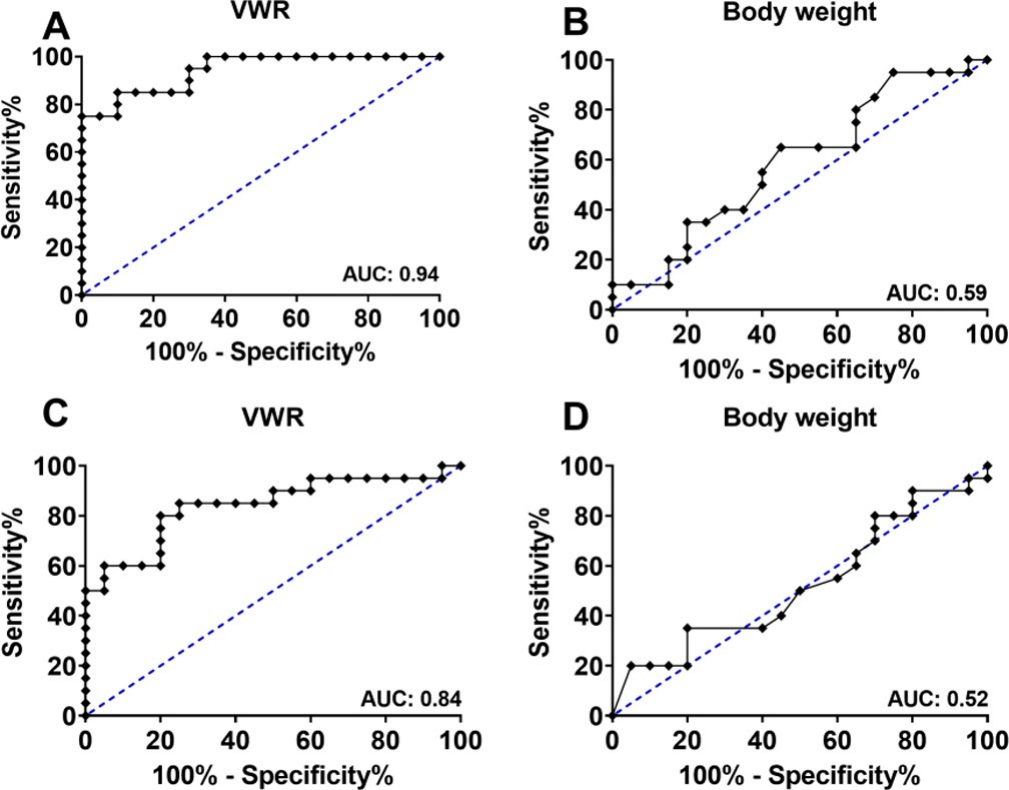
ROC curve analysis of well-being parameters. ROC curve analyses for the impact of surgery on VWR (A) and body weight (B) course and the impact of the first double injection procedure on VWR (C) and body weight (D) (n = 20).

For evaluation of the indicative quality of VWR and body weight change as parameters for impaired well-being, receiver operating characteristic (ROC) analysis was applied on post-surgical and first-injection-day data. For post-surgical VWR data, ROC curve analysis resulted in an Area under the Curve (AUC) of 94.25% (CI_95_ [0.88; 1.01], Fig 7A); the test of body weight data revealed an AUC of 59% (CI_95_ [0.4112; 0.7688]; Fig 7B). In consideration of the impact of the first IP injections without prior habituation to the procedure, ROC curve analyses were performed with data derived from day four and day five. This resulted in an AUC of 84% in VWR (CI_95_ [0.71; 0.97]; Fig 7C) and of 52% in BW (CI_95_ [0.34; 0.71]; Fig 7D).

### Cluster analysis of VWR data for severity level allocation

**Fig 8:**
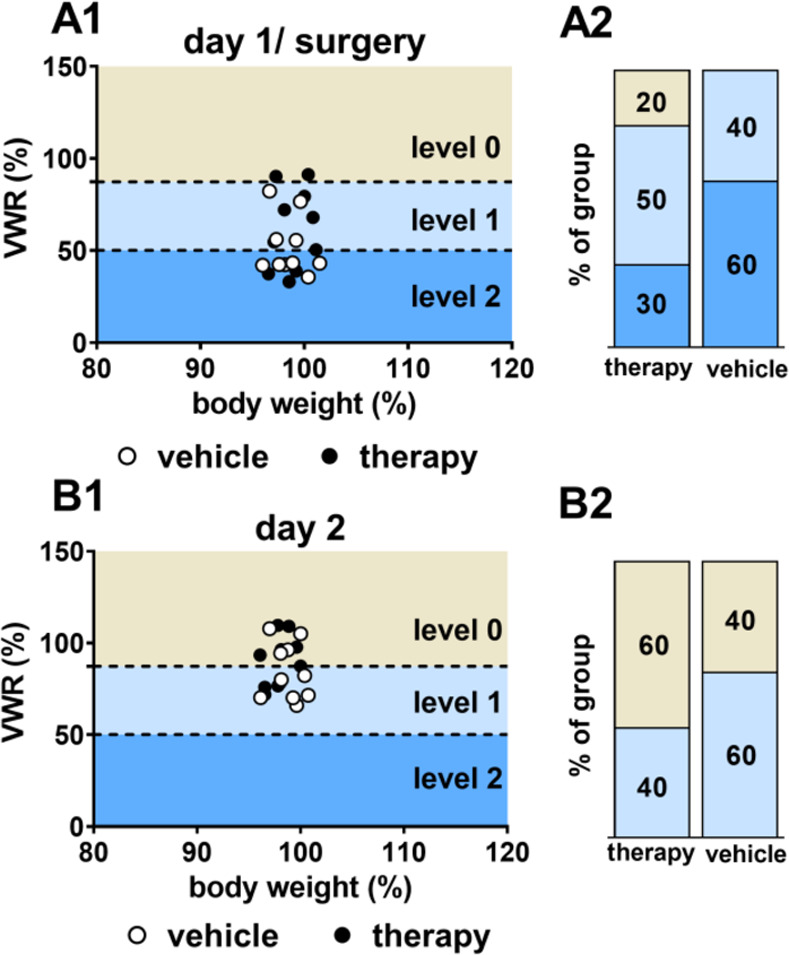
Severity level cluster for surgical intervention and recovery. Allocation to levels within cluster model for the day of surgery (A; therapy group: 30% level 2, 50% level 1, 20% level 0; vehicle group: 60% level 2, 40% level 1) and the first day of recovery (B; therapy group: 40% level 1, 60% level 0; vehicle group: 60% level 1, 40% level 0).

A previously developed cluster model [15] was applied as a tool to distinguish between severity levels using VWR data. Data are shown exemplarily for the day of surgery and the first day of recovery (day 2) as well as the early treatment period. For the day of surgery, a fraction of 60% of mice of the vehicle group and 30% of mice of the therapy group were allocated to severity level 2. On the following day, however, animals of both treatment groups were exclusively distributed in level 0 and 1 (Fig 8A and 8B).

**Fig 9:**
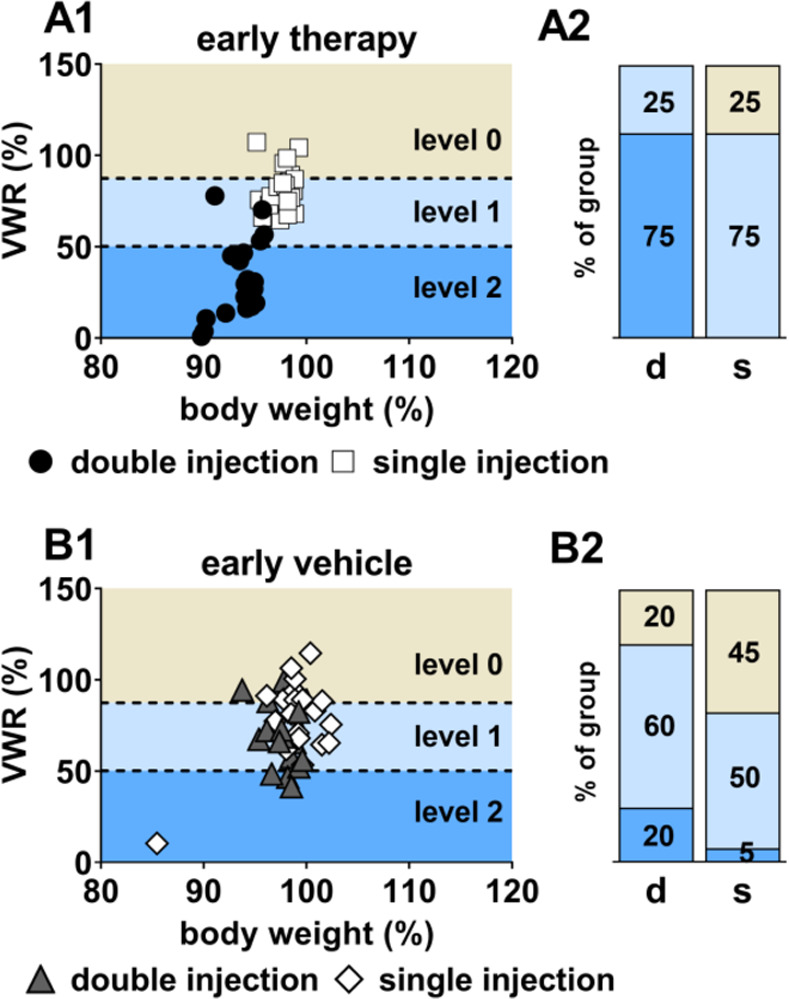
Severity level cluster for the early treatment period. Allocation to levels within cluster model for the therapy group (A) and vehicle group (B).

In the early period, a separation between single and double injection days became obvious in the therapy group: 75% of the data points of double injection days distributed into severity level 2 and 25% into level 1. In contrast, 75% of data points of single injection days distributed into level 1 and 25% into level 0 (Fig 9A). For the vehicle group, double injection days in the early period resulted in a 60% level 1-allocation. Level 2 and level 0 were represented with 20% each. Single vehicle injection in the early period led to higher percentages for level 1 and level 0 (level 0: 45%, level 1: 50%, level 2: 5%, Fig 9B).

### Tumour weights

Tumour weights at necropsy did not differ between therapy (median 0.10, CI_95_ [0.057; 00.26]) and vehicle (median 0.08; CI_95_ [−0.027; 0.48]) group (Mann-Whitney U test, U = 26; *p* = 0.71). Three animals of the vehicle group did not develop a tumour.

## Discussion

In the present study, clinical scoring, body weight changes and home-cage monitoring of VWR were used to continuously assess the well-being of mice during the post-surgical recovery phase following tumour cell injection and during the subsequent, longer phase of chemotherapy or vehicle treatment. After surgery, clinical score, body weight changes and VWR activity data indicated significant changes in the well-being of mice. During treatment phase, all parameters detected impaired well-being in the therapy group. In the vehicle group, however, an impairment was detectable solely by monitoring VWR. Further analysis showed a higher sensitivity of VWR in detecting impaired welfare compared to both body weight and clinical score. Using a previously developed cluster model based on VWR and body weight, [15], individual severity levels were classified and significant differences in the impact of single compared to double injections were identified.

In cancer research, preclinical evaluation of chemotherapeutic drugs is still performed *in vivo* to understand the complex interactions within the organism. To ensure animal welfare and high-quality research data, assessment of distress experienced by the animals is a crucial part of the experiments.

VWR behaviour is a validated, robust indicator of disturbed well-being, and was recently used to develop a cluster model to define individual severity levels in mouse models for colitis and restraint stress [15, 16]. Moreover, this behaviour has also been used to analyse adverse effects on well-being in models of depression [30], migraine [31] and to measure inflammatory pain during peripheral inflammation [19]. Furthermore, VWR was used to evaluate the recovery after surgical intervention for partial hepatectomy [17] and after surgical transmitter implantation [18]. In accordance with the literature, a reduction of VWR behaviour after surgery and cell injection was detected in the present study [17, 32], indicating impaired well-being of mice due to the surgical procedure despite analgesic treatment. By using the above-mentioned cluster model [15], it was possible to classify the severity of the experimental procedures in this study and distinguish between severity levels on the day of surgery and subsequent recovery days. Regarding the day of surgical intervention, both groups were primarily distributed to severity level 1 and 2. However, the subsequent steep increase in VWR activity indicated a rapid recovery, presuming a mild to moderate severity of this procedure. This assumption is supported by only marginally increased clinical scores and the absence of significant body weight reductions.

During subsequent treatment with galloflavin and metformin as well as during administration of the vehicle substances DMSO and PBS, animals displayed reduced VWR activity as well. The heat maps illustrate considerable changes in the activity patterns of mice not only after surgery but also during the treatment phase. In accordance with Pernold et al. [7], the activity patterns of mice during habituation (before being submitted to any intervention) showed an increase after lights were turned off, followed by a decrease after 6–8 hours and another peak in activity approximately one hour before lights were turned on. However, mice submitted to repeated intraperitoneal injections showed a decrease of activity in the first half of the night, much earlier than during baseline measurements.

In terms of severity double vs single-vehicle injections were not significantly different. However, on double-injection days a more pronounced decrease of VWR performance was detected; therefore, either DMSO or the repeated injection procedure might have a slight additional influence on VWR. Even though DMSO is generally considered to have low toxicity [33], it still leads to local irritation when used topically on the skin. Taken together, adverse effects after DMSO injection cannot be excluded and have indeed recently been corroborated in a study comparing DMSO to PBS injection [29].

However, comparing double therapy injections vs single therapy injections as well as vs double vehicle injections a significant difference was detected especially in the early and middle period of the treatment phase, indicating substance dependence and rendering galloflavin the critical factor for additionally impaired well-being in the therapy group. This is in line with a study by Kumstel et al. [34], in which galloflavin led to medication-specific suffering of the animals. However, after prolonged administration of galloflavin, the animals showed fewer signs of distress [34]. Alternatively, the repeated injection procedure may have an impact on VWR activity. This, however, cannot be deduced from the available data as a control receiving two injections without DMSO application was not part of the study design. The effect of single injections on VWR in the therapy group did not significantly vary from the effect of single injections in the vehicle group, indicating no negative effect of metformin compared to PBS.

These findings lead to the assumption of a low impact of metformin and PBS injection, and a possible slightly higher impact of DMSO, which is in line with reports in the literature [25, 29]. Additionally, a high impact of galloflavin injection was observed. These findings should be taken into consideration regarding the application of this substance in further studies and necessitate the refinement of the application route, dosage and analgesia protocol.

Apart from the influence of the respective substance and injection procedure, the handling itself could be a confounding factor. For example, in a study by Jirkof et al. [35], repeated restraint of animals for subcutaneous injections had a significant impact on activity and was therefore hypothesised to cause distress. Another study found adverse effects of handling required for intrathecal injection, which was not markedly elevated by injection procedure itself [36]. Furthermore, metamizole was administered via the drinking water over the entire duration of the experiment, but is unlikely to be a potential confounding factor. It has recently been shown that metamizole has no influence on natural behaviour, water consumption or body weight [37].

In this study, treatment of pancreatic cancer with galloflavin and metformin did not result in reduced tumour growth. Thus, the question arises whether the therapy per se failed or if any confounding factors interacted with the effects of the administered drugs or the tumour growth itself. In light of the literature supporting the positive effect of exercise on cancer in mouse models using VWR [38–40], the physical activity might have diminished or obscured the effects of chemotherapeutic treatment. Pedersen et al. [40] found a distinguished effect of exercise across five different tumour models with significant impact on endogenous factors such as interleukin-6 secretion and natural killer cell activation. These influences may have contributed to an overall decrease in tumour progression, possibly concealing drug effects. Another confounding effect might be mediated by stress due to frequent handling procedures of both therapy and vehicle treatment group. In studies investigating lymphoma in mice, it has been shown that chronic restraint stress induced tumour growth by adapting anti-tumour immune responses [41, 42]. Furthermore, in a study investigating the impact of repeated restraint stress in an orthotopic pancreatic cancer model, mice demonstrated elevated stress hormone levels accompanied by a significantly greater tumour size, which was antagonized by blockade of the HPA-axis [5]. As emotional stress is of high importance for tumour progression, stress levels in experimental animals during preclinical studies should be minimised.

Besides potentially influencing tumour growth, stress and its associated alterations in the body may also influence body weight [43]. This parameter is thus often applied for evaluation of well-being in laboratory animals. Galloflavin, the administered treatment substance has, however, been demonstrated to interfere with cell metabolism, aiming to induce deprivation of energy substrates in tumour cells. Metformin, as an anti-diabetic drug, influences the metabolism as well and has been shown to lead to weight loss in two-year-old mice [44]. As decreased body weight is generally not mentioned as a side effect in other studies, the influence is presumably negligible but might still be enhanced under exercise conditions. Studies on humans have found interactions between exercise and metformin, e.g. revealing a greater subjective feeling of fatigue under treatment [45]. Studies investigating this effect have not been performed in mice. Regarding the above-discussed effects of therapy substances, body weight change is not an ideal and reliable indicator for well-being in this study. Considering vehicle-treated animals, body weight change and clinical scoring were insufficient detect impaired well-being. Both parameters failed to capture the impact of handling and injection, which has been described as stressful [4, 46] and which was distinctly identified by monitoring VWR. ROC curve analyses corroborated these findings by showing an exceedingly higher sensitivity for VWR after surgery and after the first injection.

## Conclusion

In conclusion, the analysis of VWR behaviour as a highly sensitive indicator of distress in this study demonstrates, on both group and individual level, the impact of surgery and injection procedure on the well-being of animals. In this study, the utilization of a cluster model of VWR activity led to the characterization of thecell injection procedure to be of mild to moderate. Interestingly, repeated ip injections can be assumed to be ofmoderate severity in this model. The side effects of experimental treatments uncovered in this study should be taken into account in future studies and may lead to a change in regimens, avoiding repeated IP injections wherever possible.

## Supporting information

S1 File Overview statistics. (DOCX)

S2 File VWR values of 5-minute intervals for heat map creation. (XLSX)

S1 Fig Daytime activity patterns of baseline and post-surgical recovery phase. Heat map of the light and dark phase post-surgery. The heat map shows the VWR activity data displayed in 5- minute intervals during day- and night-time for days of baseline (day -6 to -1) and post-surgery phase (day 1 to 4). This is shown separately for the therapy group and the vehicle group. Each line represents one day of the phase (7 AM- 7 AM). For each day, the values of 5-minute intervals are summarized for each group and are colour-coded with blue representing low and red representing high VWR activity (0–400 rotations/min).
(TIF)

S2 Fig Daytime activity patterns during chemotherapy and respective vehicle application. Heat map of the light and dark phase during treatment phase. The heat map shows the VWR activity data displayed in 5-minute intervals during day- and night-time for days of baseline (day -6 to -1) and three representative periods during the experiment (early day 5 to 8, middle day 19 to 22, late day 32 to 36). This is shown separately for the therapy group and the vehicle group. Each line represents one day of the phase (7 AM-7 AM). For each day, the values of 5-minute intervals are summarized for each group and are colour-coded with blue representing low and red representing high VWR activity (0–400 rotations/min). The arrows show the days of double injections.
(TIF)
