# Supplementary material for: Voluntary wheel running behaviour as a tool to assess the severity in a mouse pancreatic cancer model
Source: PLoS One. 2021 Dec 23;16(12):e0261662. doi: 10.1371/journal.pone.0261662 (PMC8699632; doi:10.1371/journal.pone.0261662)
Supplement: S1 File — (DOCX) [file pone.0261662.s001.docx]

**S1 data:**

Levels of significance: * = *p* < 0.05, ** = *p* < 0.01, *** = *p* < 0.001, **** = *p* < 0.0001

**Clinical Score**

**Column statistics – Therapy group**

|  | Median | Mean | Std. Deviation | Std. Error of Mean | Lower 95% CI of mean | Upper 95% CI of mean |
| --- | --- | --- | --- | --- | --- | --- |
| Bsl | 0 | 0 | 0 | 0 | 0 | 0 |
| 1 | 2 | 1.6 | 1.838 | 0.5812 | 0.2853 | 2.915 |
| 2 | 0 | 0.4 | 1.265 | 0.4 | -0.5049 | 1.305 |
| 3 | 0 | 0 | 0 | 0 | 0 | 0 |
| 4 | 0 | 0.2 | 0.6325 | 0.2 | -0.2524 | 0.6524 |
| 5 | 0 | 0.4 | 1.265 | 0.4 | -0.5049 | 1.305 |
| 6 | 0 | 0.2 | 0.6325 | 0.2 | -0.2524 | 0.6524 |
| 7 | 0 | 0.1 | 0.3162 | 0.1 | -0.1262 | 0.3262 |
| 8 | 0 | 0 | 0 | 0 | 0 | 0 |
| 9 | 0 | 0 | 0 | 0 | 0 | 0 |
| 10 | 0 | 0 | 0 | 0 | 0 | 0 |
| 11 | 0 | 0.1 | 0.3162 | 0.1 | -0.1262 | 0.3262 |
| 12 | 0 | 0.2 | 0.6325 | 0.2 | -0.2524 | 0.6524 |
| 13 | 0 | 0.2 | 0.6325 | 0.2 | -0.2524 | 0.6524 |
| 14 | 0 | 0.3 | 0.6749 | 0.2134 | -0.1828 | 0.7828 |
| 15 | 0 | 0.2 | 0.6325 | 0.2 | -0.2524 | 0.6524 |
| 16 | 0 | 0 | 0 | 0 | 0 | 0 |
| 17 | 0 | 0.8 | 1.398 | 0.4422 | -0.2004 | 1.8 |
| 18 | 0 | 0.8 | 1.398 | 0.4422 | -0.2004 | 1.8 |
| 19 | 0 | 0.6 | 0.9661 | 0.3055 | -0.0911 | 1.291 |
| 20 | 0 | 1 | 1.414 | 0.4472 | -0.01167 | 2.012 |
| 21 | 0 | 0.6 | 1.265 | 0.4 | -0.3049 | 1.505 |
| 22 | 0 | 1.2 | 2.7 | 0.8537 | -0.7313 | 3.131 |
| 23 | 1 | 1.8 | 2.573 | 0.8138 | -0.04088 | 3.641 |
| 24 | 3.5 | 3.4 | 2.459 | 0.7775 | 1.641 | 5.159 |
| 25 | 3 | 2.6 | 2.119 | 0.67 | 1.084 | 4.116 |
| 26 | 2 | 2.8 | 2.15 | 0.6799 | 1.262 | 4.338 |
| 27 | 0 | 0.5 | 0.8498 | 0.2687 | -0.1079 | 1.108 |
| 28 | 0 | 0.6 | 0.9661 | 0.3055 | -0.0911 | 1.291 |
| 29 | 0 | 0.6 | 0.9661 | 0.3055 | -0.0911 | 1.291 |
| 30 | 0 | 0.9 | 1.197 | 0.3786 | 0.04356 | 1.756 |
| 31 | 0 | 0.2 | 0.6325 | 0.2 | -0.2524 | 0.6524 |
| 32 | 0 | 0.2 | 0.6325 | 0.2 | -0.2524 | 0.6524 |
| 33 | 0 | 0.3 | 0.6749 | 0.2134 | -0.1828 | 0.7828 |
| 34 | 0 | 0.8 | 1.398 | 0.4422 | -0.2004 | 1.8 |
| 35 | 0 | 0.4 | 0.8433 | 0.2667 | -0.2032 | 1.003 |
| 36 | 0 | 0.4 | 0.8433 | 0.2667 | -0.2032 | 1.003 |
| 37 | 0 | 0.4 | 0.8433 | 0.2667 | -0.2032 | 1.003 |

**Clinical Score**

**Column statistics - Vehicle group**

|  | Median | Mean | Std. Deviation | Std. Error of Mean | Lower 95% CI of mean | Upper 95% CI of mean |
| --- | --- | --- | --- | --- | --- | --- |
| Bsl | 0 | 0 | 0 | 0 | 0 | 0 |
| 1 | 0 | 0.9 | 1.197 | 0.3786 | 0.04356 | 1.756 |
| 2 | 0 | 0 | 0 | 0 | 0 | 0 |
| 3 | 0 | 0 | 0 | 0 | 0 | 0 |
| 4 | 0 | 0 | 0 | 0 | 0 | 0 |
| 5 | 0 | 0.2 | 0.6325 | 0.2 | -0.2524 | 0.6524 |
| 6 | 0 | 0 | 0 | 0 | 0 | 0 |
| 7 | 0 | 0.8 | 2.53 | 0.8 | -1.01 | 2.61 |
| 8 | 0 | 1.5 | 4.403 | 1.392 | -1.65 | 4.65 |
| 9 | 0 | 0.4444 | 0.8819 | 0.294 | -0.2335 | 1.122 |
| 10 | 0 | 0.4444 | 0.8819 | 0.294 | -0.2335 | 1.122 |
| 11 | 0 | 0 | 0 | 0 | 0 | 0 |
| 12 | 0 | 0 | 0 | 0 | 0 | 0 |
| 13 | 0 | 0 | 0 | 0 | 0 | 0 |
| 14 | 0 | 0 | 0 | 0 | 0 | 0 |
| 15 | 0 | 0 | 0 | 0 | 0 | 0 |
| 16 | 0 | 0 | 0 | 0 | 0 | 0 |
| 17 | 0 | 0 | 0 | 0 | 0 | 0 |
| 18 | 0 | 0.1111 | 0.3333 | 0.1111 | -0.1451 | 0.3673 |
| 19 | 0 | 0 | 0 | 0 | 0 | 0 |
| 20 | 0 | 0.6667 | 1.414 | 0.4714 | -0.4204 | 1.754 |
| 21 | 0 | 0 | 0 | 0 | 0 | 0 |
| 22 | 0 | 0 | 0 | 0 | 0 | 0 |
| 23 | 0 | 1.444 | 3.127 | 1.042 | -0.9591 | 3.848 |
| 24 | 0 | 0.2222 | 0.6667 | 0.2222 | -0.2902 | 0.7347 |
| 25 | 0 | 0.3333 | 0.7071 | 0.2357 | -0.2102 | 0.8769 |
| 26 | 0 | 0.4444 | 1.333 | 0.4444 | -0.5804 | 1.469 |
| 27 | 0 | 0.6667 | 1 | 0.3333 | -0.102 | 1.435 |
| 28 | 0 | 0.3333 | 0.7071 | 0.2357 | -0.2102 | 0.8769 |
| 29 | 0 | 0 | 0 | 0 | 0 | 0 |
| 30 | 0 | 0.2222 | 0.6667 | 0.2222 | -0.2902 | 0.7347 |
| 31 | 0 | 0 | 0 | 0 | 0 | 0 |
| 32 | 0 | 0 | 0 | 0 | 0 | 0 |
| 33 | 0 | 0 | 0 | 0 | 0 | 0 |
| 34 | 0 | 0 | 0 | 0 | 0 | 0 |
| 35 | 0 | 0.1111 | 0.3333 | 0.1111 | -0.1451 | 0.3673 |
| 36 | 0 | 0.5556 | 1.333 | 0.4444 | -0.4693 | 1.58 |
| 37 | 0 | 0 | 0 | 0 | 0 | 0 |

**Clinical score**

| **Wilcoxon signed rank test of clinical score data for in-group comparison to baseline** |
| --- |

| **Mann-Whitney *U* test for comparison between groups** |
| --- |

**Body weight:**

| **Linear mixed-effects model for comparison within groups to baseline**  **** |
| --- |

Post-hoc test: Dunnett’s multiple comparisons test, see Table 2.2.

| **Dunnett’s multiple comparisons test for comparison of body weight change to baseline** |
| --- |

| **Linear mixed-effects model for comparison of body weight change between groups** |
| --- |

Correction for multiple comparisons: Bonferroni’s adjustment, see Table 2.4.

| **Bonferroni’s adjustment for multiple comparisons of body weight change between groups** |
| --- |

**VWR:**

| **Linear mixed-effects model for comparison of VWR change to baseline** |
| --- |

Posthoc test: Dunnett’s multiple comparisons test, see Table 3.2.

| **Dunnett’s multiple comparisons test for comparison of VWR change to baseline** |
| --- |

| **Linear mixed-effects model for comparison of VWR change between groups** |
| --- |

Multiple comparisons adjustment: Bonferroni, see Table 3.4.

| **Bonferroni’s adjustment for multiple comparisons of body weight change between groups** |
| --- |

| **Early period: two-way repeated measures ANOVA for comparison between group and injection frequency** |
| --- |

Posthoc test: Tukey’s multiple comparisons test, see Table 3.6.

| **Early period: Tukey’s multiple comparisons test for comparison between group and injection frequency** |
| --- |

| **Intermediate period: two-way repeated measures ANOVA for comparison between group and injection frequency** |  |
| --- | --- |

Posthoc test: Tukey’s multiple comparisons test, see Table 3.8.

| **Intermediate period: Tukey’s multiple comparisons test for comparison between group and injection frequency** |  |
| --- | --- |

| **Late period: two-way repeated measures ANOVA for comparison between group and injection frequency** |
| --- |

Posthoc test: Tukey’s multiple comparisons test, see Table 3.10.

| **Late period: Tukey’s multiple comparisons test for comparison between group and injection frequency** |  |  |
| --- | --- | --- |

**Tumour weights:**

| **4.1: Table: Mann-Whitney *U* test of tumour weights** |
| --- |
