# Supplementary figures and images for: Voluntary wheel running behaviour as a tool to assess the severity in a mouse pancreatic cancer model
Source: PLoS One. 2021 Dec 23;16(12):e0261662. doi: 10.1371/journal.pone.0261662 (PMC8699632; doi:10.1371/journal.pone.0261662)

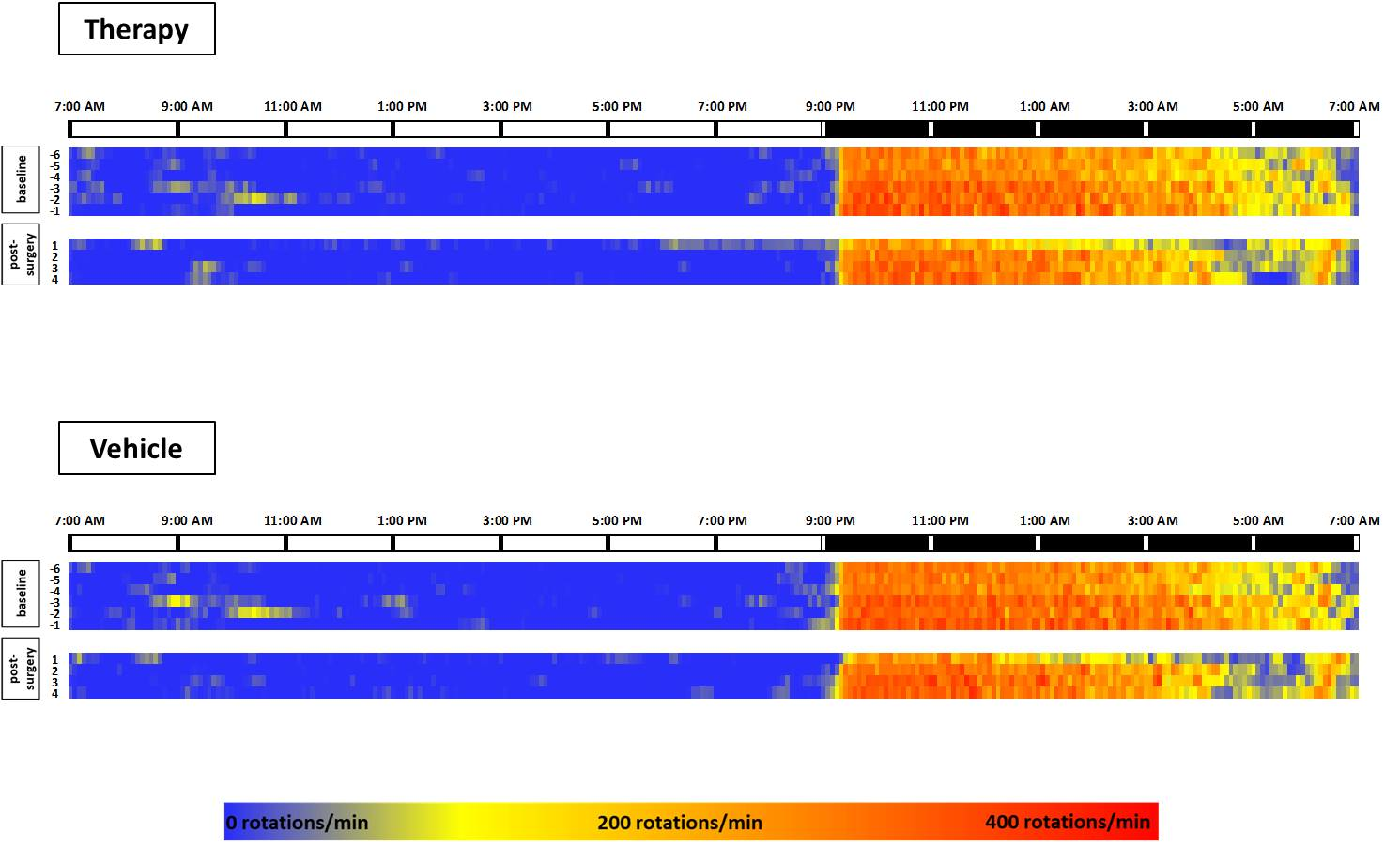

Supplement: S1 Fig — Heat map of the light and dark phase post-surgery. The heat map shows the VWR activity data displayed in 5- minute intervals during day- and night-time for days of baseline (day -6 to -1) and post-surgery phase (day 1 to 4). This is shown separately for the therapy group and the vehicle group. Each line represents one day of the phase (7 AM- 7 AM). For each day, the values of 5-minute intervals are summarized for each group and are colour-coded with blue representing low and red representing high VWR activity (0–400 rotations/min). (TIF) [file pone.0261662.s003.tif]

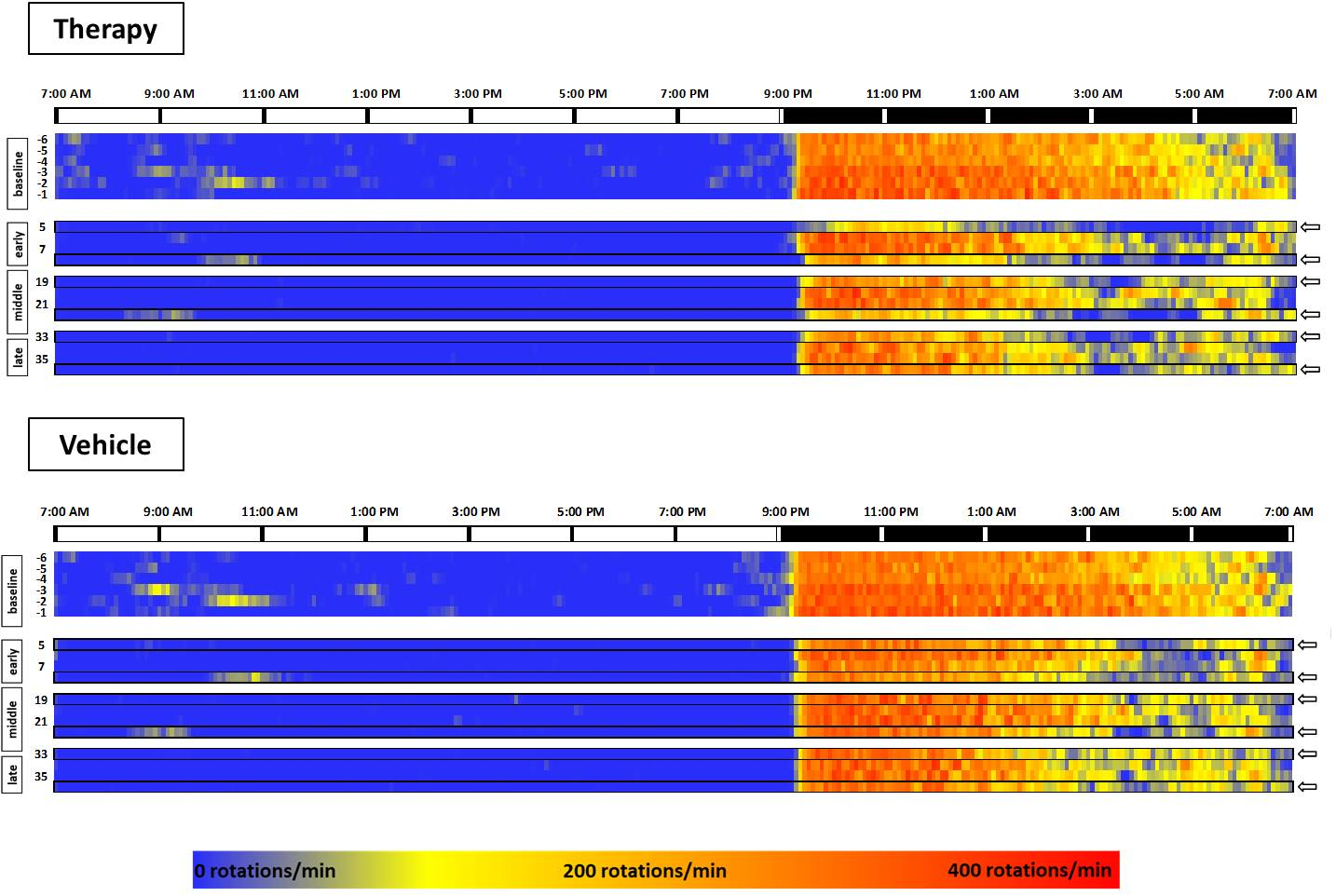

Supplement: S2 Fig — Heat map of the light and dark phase during treatment phase. The heat map shows the VWR activity data displayed in 5-minute intervals during day- and night-time for days of baseline (day -6 to -1) and three representative periods during the experiment (early day 5 to 8, middle day 19 to 22, late day 32 to 36). This is shown separately for the therapy group and the vehicle group. Each line represents one day of the phase (7 AM-7 AM). For each day, the values of 5-minute intervals are summarized for each group and are colour-coded with blue representing low and red representing high VWR activity (0–400 rotations/min). The arrows show the days of double injections. (TIF) [file pone.0261662.s004.tif]
